# COVID-19 pandemic: ten research questions Africa must answer for itself

**DOI:** 10.4314/gmj.v54i4s.17

**Published:** 2020-12

**Authors:** Evelyn Y Bonney, Helena Lamptey, Peter Puplampu, George B Kyei

**Affiliations:** 1 Virology Department. Noguchi Memorial Institute for Medical Research, College of Health Sciences, University of Ghana. Off Akilagpa Sawyerr Road. PO Box LG 581, Legon, Accra, Ghana; 2 Immunology Department. Noguchi Memorial Institute for Medical Research, College of Health Sciences, University of Ghana. Off Akilagpa Sawyerr Road. PO Box LG 581, Legon, Accra, Ghana; 3 Department of Medicine, University of Ghana Medical School. College of Health Sciences, University of Ghana. PO Box GP 4236, Accra, Ghana; 4 Medical and Scientific Research Directorate, University of Ghana Medical Centre, UG Medical Centre. Post Office Box LG 25, Legon, Accra, Ghana; 5 Department of Medicine, Washington University School of Medicine, 660 S. Euclid Avenue, St. Louis, MO, USA

**Keywords:** COVID-19, research, development, Africa

## Abstract

The COVID-19 pandemic is spreading through Africa and governments are making frantic efforts to control spread, hospitalizations and deaths. While control measures are being taken, research into the biomedical and socio-cultural aspects of the pandemic, relevant to the African population, should not be ignored. It should not be assumed that research performed in Asian, American and European populations will always be applicable to Africa. Rather, research should be done in Africa to answer questions peculiar to the epidemic on the continent and help inform international guidelines. National guidelines for treatment and prevention, patient recoveries and discharge, and public health control measures should be based on research performed in the appropriate context for them to be effective and robust. Urgent research is needed in viral immunology and shedding, treatment and prevention trials, protection of healthcare personnel, and antimicrobial use among others. In this article, we propose ten research questions that when answered in a timely manner by scientists in Africa, will enhance Africa's response to the pandemic.

**Funding:**

GBK is supported by a fellowship from the European Developing Countries Clinical Trials Fellowship as part of the EDCTP (2) program. The funder had no role in the preparation of this manuscript.

## Introduction

The COVID-19 pandemic caused by the severe acute respiratory syndrome corona virus -2 (SARS-CoV-2) continues to cause havoc with over 17 million confirmed infections worldwide at the time of writing.^1^ Africa came to the pandemic late and infections and deaths are currently rising on a daily basis around the continent. Modeling by the World Health Organization (WHO) predicts up to 44 million cases and 190,000 deaths in Africa in the first year of the pandemic unless drastic measures are taken.^2^ While African governments are putting in place measures to slow the pandemic, it is crucial that important research questions are not ignored and seeded to other countries. Funds should be earmarked for meaningful research, and Africa's research infrastructure should be mobilized to answer questions relevant to the continent.

We cannot assume that research findings in Europe or America will always be applicable to the African population given the different genetics, environment and socio-economic situations. In this article, we discuss ten research questions that should be answered in the African context to help overcome the pandemic.

### Question 1: What is the quality of antibodies produced and how long do they last?

Almost all immune competent people infected with the SARS-CoV-2 virus will produce some antibodies. In general IgM antibodies are produced 1–2 weeks after the onset of illness followed by IgG. While IgM is shortlived, IgG tend to persist. For SARS-CoV-2 however, it seems IgG also appear by the time IgM is detected.

The question of whether durable antibodies are produced by SARS-CoV-2 infected patients is now beginning to be elucidated. There are few reasons why these studies, though being performed in other parts of the world, should also be performed in Africa, and for that matter Ghana.

First, the magnitude of antibody produced, and how long they last, differ among hosts. These differences are usually genetic or environmental depending on how the person's immune system has been educated over time. Therefore, there is the need for longitudinal studies to determine if different categories of patients produce antibodies and for how long. Such groups include asymptomatic/pauci-symptomatic patients, sick and recovered patients, stratified into age groups such as children, adolescents, adults and the elderly. This will help determine who is still at risk after they recover from COVID-19. Results coming out of Europe and the China indicate that antibodies to SARS-CoV-2 may not last.^3^,^4^ While the antibodies seem to decay rapidly, it remains to be determined if the residual antibodies are protective.

The second reason to determine antibody titers in a wide variety of patients is to identify which patients produce good neutralizing antibodies that can bind and disable the virus. Such patients could be asked to donate serum that can be used to treat acutely ill patients, like those in the intensive care units. These studies are important because the fact that a recovered patient has antibodies does not necessarily mean that those antibodies are neutralizing. It is more useful when neutralization assays are performed in the laboratory to find breadth of those antibodies with the best neutralizing capacity. Furthermore, the technology now exists to isolate B lymphocytes from such patients and manipulate them to produce these good neutralizing monoclonal antibodies in large quantities.

Third, since most patients with SARS-CoV-2 infection are asymptomatic and therefore may not have come into contact with the healthcare system to be tested, it is important to perform antibody surveillance studies in the population to determine what percentage of the population have been exposed and may have immunity against the virus. If we determine that immunity does not last especially among asymptomatic individuals, it will have implications for how long to continue measures like social distancing and the wearing of masks. Another crucial related question is how long the virus is shed in stool and whether that is important for transmission.^5^ Research in this area is very limited but it is a question that must be answered in Africa where sanitation is usually poor. Can SARS-CoV-2 be transmitted feco-orally? Can flies transfer the virus from one place to the other through fecal matter?

### Question 2: What is the duration of viable virus shedding among Africans?

The duration of viral shedding is at the core of viral spread and therefore must be accurately determined. Studies from other parts of the world show that viral shedding may last for about 14 days or more. Current WHO guidelines recommend that patients could be considered non-infectious (for the most part) 14 days after their first test. Ghana and other African countries have adopted these recommendations. It is instructive that these recommendations are based on studies performed in Asia, Europe and America. Indeed out of the 43 references that informed this guideline, none was performed in African patients, yet it seems the recommendation was tailored for Africa where testing capacity is limited.^6^ The standard polymerase chain reaction (PCR) test only determines if viral fragments are present (positive) or not (negative), but cannot tell if the person being tested is infectious. Infectivity can only be determined by viral cultures. It is important to do systematic studies in African patients of all ages, symptomatic and asymptomatic to determine by quantitative PCR and viral cultures, how long viable virus is shed by infected individuals. Such knowledge will give more reliable data for policies on recoveries. We may even find that 14 days is too long for the vast majority of Africans who are asymptomatic. Even a reduction from 14 to 10 days will save huge amounts of resources in isolation and contact tracing costs and enable infected individuals to return to work early. The current data from Africa is not strong for the policies being implemented. What do we do with healthcare personnel (HCP) for example? Should they be considered recovered after 14 days and go back to work? Is it worth the small risk that they may be able to infect some of their vulnerable patients? Or should HCP document negative tests before going back to work?

### Question 3: What clinical trials will give the most impact in Africa?

Numerous clinical trials for treatment and potential vaccines are ongoing for COVID-19 around the world. Trials have shown the usefulness of remdesivir and dexamethasone in critically ill patients and the probable ineffectiveness of chloroquine in hospitalized patients.^7^ Other antivirals such as sofosbuvir/ daclatasvir, favipiravir and merimepodib are being tried.^8^ Many African countries are engaged in the WHO-led SOLIDARITY trial which is testing various agents including hydroxychloroquine, HIV protease inhibitor lopinavir/ritonavir and remdesivir. Already hydroxychloroquine and lopinavir/ritonavir have been discontinued for being ineffective.^9^ The African Covid-19 Critical Care Outcomes Study (ACCCOS) is collecting data on critically ill and intensive care unit (ICU) patients across the continent to determine their characteristics, survival and treatments. These are commendable initiatives that should be supported. Aside from participating in these trials, Africans must also design observational studies to follow patients who are admitted or isolated to detail their clinical characteristics more closely. In so doing, we may discover presentations of COVID-19 that are unique to particular environments. Ethical reviews should be effective and prompt at the institutional and regulatory levels to ensure that we can participate in these trials on time. The question is should Africa be proactive in participating in these trials or should we wait till treatments are proven? We argue that full participation in prevention and treatment trials is essential. Participation will let us discover early if these interventions work in Africa and ensure that countries do not spend scarce resources to buy vaccines or treatments that may not work for patients on the continent in the long run. Particularly, Africa must be aggressive in participating in prevention trials designed for HCPs. We cannot afford to have our scarce HCPs infected resulting in long periods off the job or sometimes even death. Therefore, trials that seek to prevent COVID-19 should be priority. There are indications that BCG vaccination and measles/mumps vaccinations or re-vaccinations may be protective.^10^,^11^ Africans should design and conduct trials using some of these relatively cheap interventions. If they work, they will be more accessible in Africa, compared to expensive interventions that are not likely to reach majority of the population.

### Question 4: Should we be screening African herbal products for COVID-19?

A large proportion of Africans depend on herbal medicine to treat all manner of ailments. There are claims of herbal products efficacy for COVID-19 most famously in Madagascar. Although Madagascar's claim has not stood the test of time, it does not mean we should ignore all the herbal medicine that abound in Africa. Many African countries have herbal repositories that can be screened to identify products that may be effective against SARSCoV-2. In Ghana, the Mampong Centre for Plant Medicine Research has a large collection of herbal products that could be screened. The problem with SARS-CoV-2 is that screening of drugs is not a trivial matter. It requires complex infrastructure for cell culture and biosafety level 3 facilities. These facilities are few on the continent. In Ghana, these can only be found at the Noguchi Memorial Institute for Medical Research (NMIMR) and Kumasi Center for Collaborative Research (KCCR). Some countries have no such facilities. That said, many countries in Africa are members of the Emerging and Dangerous Pathogens Laboratory Network (EDPLN), which consist of laboratories with biosafety level 3 (BSL3) facilities capable of handling dangerous pathogens like Ebola, Marburg and SARS-CoV-2. EDPLN facilities could come together and screen compounds from neighboring countries without the requisite facilities. Where the infrastructure exists for such screening activities, what is needed is funding for the necessary personnel, reagents and consumables needed to undertake these tasks. African governments should invest in these, because effective herbal treatment for COVID-19 from Africa is likely to be less expensive and accessible to many on the continent.

### Question 5: How is COVID-19 affecting antimicrobial use and resistance?

Antimicrobial resistance (AMR) is a slow pandemic on its own accounting at least 700,000 deaths annually, with estimated toll of 10 million death per year by 2030.^12^The COVID-19 pandemic could contribute substantially to this dire situation in two ways: First is prescription of unneeded antibiotics followed by development of resistance from continuous use of antimicrobial hand washes. The WHO has already sounded the alarm about the increasing use of antibiotics during the pandemic, noting that even people with mild to moderate COVID-19 symptoms are being given prophylactic antibiotics.^13^. In addition, a recent meta-analysis showed that though bacterial infections play a role in less than 10% of COVID-19 infections, antibiotics (mainly broad spectrum) were used in at least 72% of patients.^14^ This issue becomes even more critical in Africa where (i) delays in COVID-19 diagnosis due to limited laboratory capacity could result in prolonged use of antibiotics for the pneumonia syndrome and (2) cultures may not be available and therefore physicians may continue to use antibiotics even after SARSCoV-2 is confirmed.

Currently, there are no studies showing which bacterial organisms (if any) are commonly isolated in COVID-19 patients in Africa. These studies are urgently needed to give prescribers confidence to pick narrow spectrum antibiotics when required and stop antibiotics faster when SARS-CoV-2 is confirmed. Secondly, longitudinal studies are needed, especially among HCP to determine if frequent handwashing with antibacterial soaps changes their flora to select for resistant organisms that they could in turn transfer to vulnerable patients.

### Question 6: How is COVID-19 affecting care for noncommunicable and other communicable diseases?

COVID-19 has dramatically reduced outpatient visits around the world, and Africa has not been spared. One model estimates that more people will die in Africa from HIV and its comorbidities due to COVID-19-related interruptions in the health system.^15^ How about care for hypertension, diabetes, heart diseases, and communicable diseases like tuberculosis? For patients with HIV and TB, skipping clinic put them at risk for the development of drug resistance, treatment failure and death.

For those with NCDs, complications like heart failure, renal failure, myocardial infarctions, and stroke are more likely. Several urgent research questions come to mind. First, what can be done to encourage patients to come to clinic? How do caregivers feel about seeing the large number of patients they are used to seeing at the risk of contracting COVID-19 from asymptomatic patients? Are there ways to use an appointment system to decongest clinics? How about telemedicine and delivery of medications to patients? With the high penetrance of cell phone use in Africa, some of these solutions may be possible. However, the research must be done to ensure that solutions fit a particular demographic or community. Some of these innovations, if developed in the COVID-19 era, could become the standard of care afterwards.

### Question 7: How do we protect healthcare personnel from infections on the job?

Healthcare personnel are a scarce resource in Africa. Data on how many HCPs in Africa are infected is rare but there are indications that the numbers are increasing. For instance, the Ghana Health Service reported recently that at least 2000 HCPs in Ghana have been infected.^16^ While these infections could have been acquired off the job, they most likely were acquired on the job. Research is needed to determine the factors responsible for HCPs acquisition of COVID-19 so that appropriate remedial measures could be taken. Is it because of the unavailability of personal protective equipment (PPE)? Where PPE are available, are they donned and doffed properly? Are patients' rooms cleaned properly? How about infection control in hospitals? Do hospitals have robust infection control programs? How about training for HCPs and other hospital staff in infection control? Are there interventions that could be put in place to reduce the risk of acquiring COVID-19 during patient encounters? Some have suggested (without much evidence) that mouthwashes like chlorhexidine or hydrogen peroxide, if used prior to dental and surgical encounters could prevent transmission to HCPs and vice versa. Urgent clinical trials bolstered with good laboratory analysis are needed to study these inexpensive interventions. One study in two patients in Korea showed undetectable virus (by quantitative PCR) in the throat more than 2 hours after a 30 second rinse with chlorhexidine.^17^ Therefore, there is good reason to do some of these trials in Africa. If such interventions work, caregivers and patients alike will have more confidence in coming back to clinic.

### Question 8: What strains of SARS-CoV-2 are circulating in Africa?

Viruses mutate on a regular basis and change over time. Changes in amino acids could result in changes in virulence, infectivity and response to drugs. Therefore, African scientists must constantly perform surveillance and sequence the viral genome to ensure that sequences for circulating strains are up to date in the database. Such knowledge is also critical for vaccine design and whether a particular vaccine will be effective. Additionally, with reports of waning immunity, changes in viral sequence will make it easier for reinfections to occur in the population. Infrastructure for sequencing, and the expertise to analyze these sequences are now available in many African countries including Ghana. Through collaborations, countries without sequencing facilities can send samples to their neighbors who can help undertake these studies. This will provide a comprehensive view of circulating viruses on the continent and inform efforts in countries like South Africa to produce vaccines for COVID-19.

### Question 9: How do we overcome stigma?

Stigmatization for acquiring the virus is becoming a hindrance to fighting the pandemic in Africa .^18^ The fear of stigma prevents people who are having symptoms from coming forward to be tested and treated. This may lead to more deaths and more severe illness. In addition, this results in more infections as infected persons may be in the community without isolation. Several questions arise. What are the specifics of a community that gives rise to stigma? Why is it that the culture of wearing masks seems less accepted in Africa and America than in Asia and Europe for example? Is law enforcement the best way to encourage mass adoption of mask wearing? What kinds of education programs are effective for social distancing and mask wearing? The answers to these questions may be different for different communities in Africa or even different regions of the same country. Social scientists, implementation scientists and psychologists in Africa should propose studies to answer these important questions.

### Question 10: Why is the trajectory of deaths different in different countries?

Africa, where social distancing is more difficult seems to have been spared large number of deaths from the COVID-19 pandemic. Is this due to social factors such as the younger population and lack of nursing homes, or genetic and immune system differences? Others have hypothesized about malaria, BCG vaccination and latent tuberculosis as possible protective measures^19^. All of these are worthy of serious investigation. The case fatality rate is very different among countries. Are these real differences or artifacts of measurement? Is the low case fatality rate due to lack of testing and avoidance of the healthcare system? In Ghana for instance, people who get sick and die at home or on arrival at the hospital, if it is due to COVID-19, will not be counted in the national database. The way to determine if COVID-19 is contributing a lot more deaths than we are measuring is to compare with the number of deaths within the same period last year.

In Africa, most countries do not have this basic statistic. In South Africa where data is available, their medical research council reports that the virus is causing much more deaths than the official figures .^20^

## Conclusion

Over the past decade research capacity in Africa has improved, thanks to new models in north-south cooperation, investments in research infrastructure and emphasis on human capacity building. The COVID-19 pandemic is a good test to see whether these initiatives are yielding the expected results. To succeed, Africa must make prudent use of its resources. In many African countries, COVID-19 testing is done in academic research centers because the national reference laboratories are not well equipped. Ironically, this model will make Africa fall behind in COVID-19 research. The job of an academic research center in a pandemic like this is not routine diagnosis, but rather meaningful research that will help end the pandemic. Research laboratories should develop protocols and train clinical laboratories to do the routine work so that they can concentrate on answering key research questions. African governments should use this opportunity to equip their reference laboratories to do molecular diagnoses like the one required for SARS-CoV-2. This will ensure that when a pandemic hit, university research laboratories will have the breathing room to do research at pace with the disease. As governments in Africa make provision for COVID-19, research should be high on their agenda. Donors like the EDCTP, Wellcome Trust, UK NIHR and others are funding research in Africa, which is commendable. However, COVID-19 research on the continent should not be left to donors alone, who often will fund what interests them. African governments should budget for research as part of the COVID-19 response and academics and other stakeholders should lobby officials for research funds. Africa has the right facilities and personnel (scientists with relevant training and skills) to answer all the research questions raised here. With the appropriate collaborations and adequate funding these questions will be answered rigorously to help end the pandemic faster on the continent.
